# The effect evaluation of advanced penlight

**DOI:** 10.1371/journal.pone.0205978

**Published:** 2018-11-07

**Authors:** Piao-Yi Chiou, Chih-Yin Chien, Yi-Horng Lai, Chang Feng Chun

**Affiliations:** 1 Department of Nursing, National Taipei University of Nursing and Health Sciences, Taipei City, Taiwan; 2 Department of Nursing, Mackay Medicine, Nursing and Management College, Taipei City, Taiwan; 3 Department of Health Care Administration, Oriental Institute of Technology, New Taipei City, Taiwan; 4 Department of Optometry, Mackay Medicine, Nursing and Management College, Taipei City, Taiwan; Tokai University, JAPAN

## Abstract

Pupil diameter measurement is crucial for physical assessment and disease monitoring in a health and nursing care situation. A general penlights (GPLs) is frequently used and allow for an approximate and indirect measurement of the pupil diameter. Health caregivers or nurses generally have less confidence in the value of the pupil diameter measured using the GPL. The Advanced Penlight (APL) is a new device designed for accurate measurement of the pupil diameter. The purpose of the presented research was to compare the accuracies and operational times of the pupil diameter measurements by means of the GPL and APL. One-group post-test and single-blind study designed was used in this study. The innovation of the APL is the addition of a perspective measurement ruler (PMR) attached to one side of the penlight that allows precise measurement of the pupil diameter before and after pupillary contraction. The PMR can be rotated by any angle for adaptation to the measurement conditions. After standard pupil diameter measurements by a refractometer (RM) were performed on a subjects, ninety study participants measured the pupil diameters of the same subject separately by the GPL and APL. A self-administered questionnaire was used to assess the opinions of the participants after using the GPL compare to the APL. The mean age of the participants was 20.01 (SD = 0.47) years and 83% of them were female senior nursing students. There were no statistically significant differences between the average values of pupil diameters measured by the APL and the RM. Compared to the GPL, the pupil diameter measured by APL was much similar to the RM measurement. The average operational time was 8.72 seconds shorter (t = -3.81, p = 0.001) for the APL measurement compared to the GPL measurement. The average scores of convenience and confidence on pupil diameter measurements of questionnaire were higher for the APL compared to the GPL. The APL can increase the accuracy and save operating time of pupil diameter measurement and thereby promote the quality of health assessment and nursing care practice.

## Introduction

Pupil size and reactivity assessment is a regular health and nursing care in general ward, emergency room (ER), and intensive care unit (ICU) [1–2]. Measuring pupillary contraction and change of size after light stimulation can serve as a window to view the brain and evaluate the function of the autonomous nervous system. In addition, pupillometry can be used for early diagnosis of related diseases, to assess the severity of disease, to decide on treatment and nursing care strategy, and to predict the outcome of disease [1–8]. Measuring pupil diameter is also applied to study recognition memory. It is found that the pupils become significantly enlarged when the subjects see the old and familiar items [9]. Under normal conditions, the pupil diameter should be the same in both eyes. The pupil diameter ranges from 1.5 to 6.0 mm. Light stimulation of the pupil causes its contraction, which is also known as the pupil reflex [10].

A penlight provides a source of light and has become the most common used tool to assess the pupil diameter. Asymmetry of pupil constriction in response to light means one pupil constricts and the other remains dilated or constricts more slowly. It may indicate dynamic anisocoria or a Marcus Gunn pupil, a relative afferent pupillary defect (RAPD), or temporal lobe herniation in the brain [11–13]. The pupil measurement ruler is usually attached to the side of general penlights (GPL). The pupil diameter can be measured only after removing the GPL and placing the ruler close to the eye to approximately and indirectly estimate the pupil diameter. The use of the GPL not only extends the evaluation time but also reduces the accuracy of pupil diameter measurement. A number of studies have found the the effects of using different equipment on the outcomes of pupil diameter measurements [14–17]. The accuracy of a pupil diameter measurement and operation time required to perform it are the important characteristics of all new instruments or systems. However, the bulky designs of the refractometer or computer system used for pupil diameter measurement utilized in the above mentioned studies complicate their use in clinical conditions and on patients in critical and intensive care environment. Although the GPL is convenient to use, it has been observed that health caregiver or nurses generally have less confidence in the value of the pupil diameter measured using the GPL. Hence, it is important to redesign the GPL to solve the difficulties and promote the accuracy of pupil diameter measurement in a patient care situation. The purpose of this study is to compare the accuracy and the operational time of pupil diameter measurement by the GPL and a new design penlight.

## Methods

### The design of the advanced penlight

**Fig 1:**
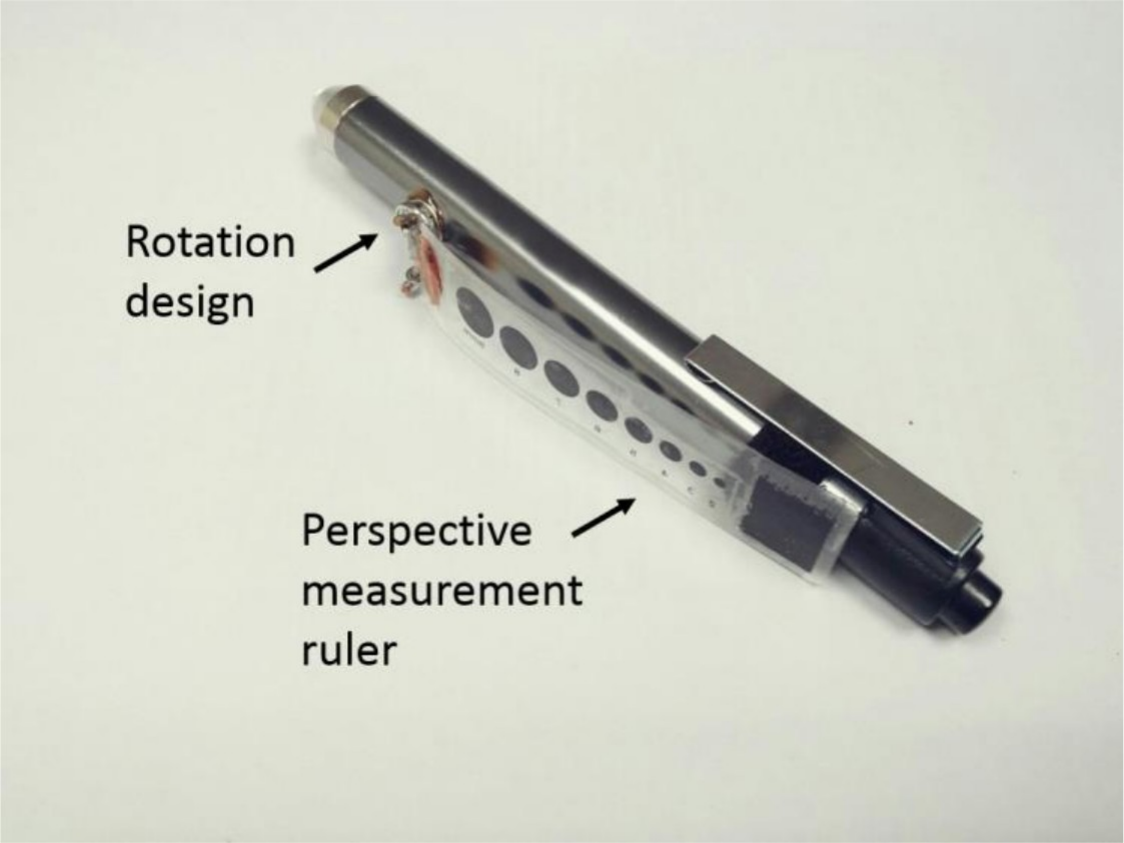
The model of APL.

**Fig 2:**
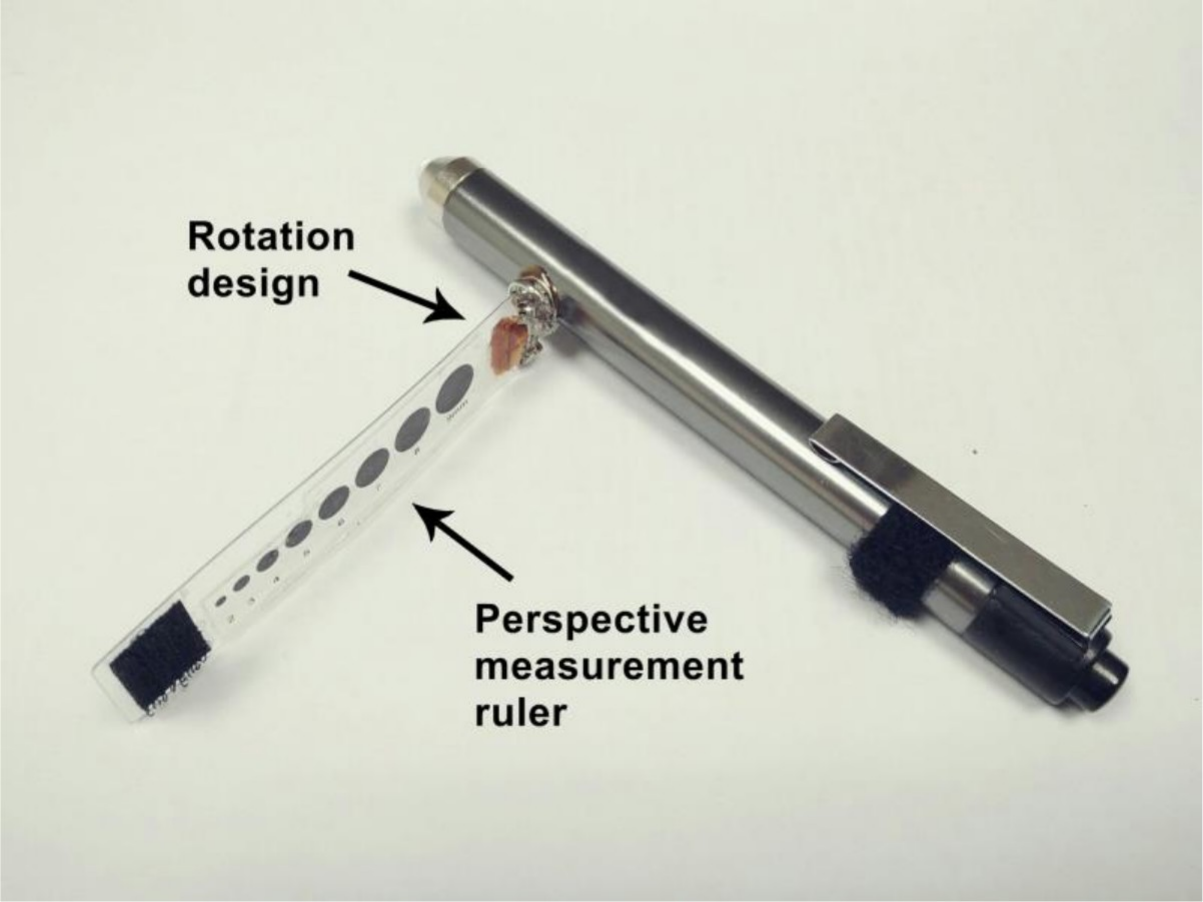
3D printing of APL.

To improve the accuracy and convenience of pupil diameter measurement, our research team designed an advanced penlight (APL) (Fig 1). There are two innovations in the design of the proposed APL. One type has a standard pupil size of perspective measurement ruler (PMR). The PMR and rotary design were made by following several steps. Eight sizes of standard pupil diameter (from 2 mm to 9 mm) was printed on a transparent plastic plate as a PMR, which is 5 cm long and 1 cm wide. There is a two-piece metal snap to attach the PMR to the penlight. The bottom part of the snap is attached to the top of the PMR. The top part of the metal snap is than attached to the bottom side of the plate. Next, the hook side of the fastener, which is made by Velcro, was stuck to the bottom of the PMR. The loop side of fastener was stuck at the opposite bottom side of the penlight to adhere the PMR. The PMR can be placed close to the eyes and directly measure the precise value of the pupil diameter before and after pupillary contraction. The metal snap is a rotary design used to fix the PMR and measure the pupil diameter at the desired degrees. The bulb voltage of APL is 2.2 V / 0.25 A. Fig 2 were 3D printing of APL.

### Experimental design

**Fig 3:**
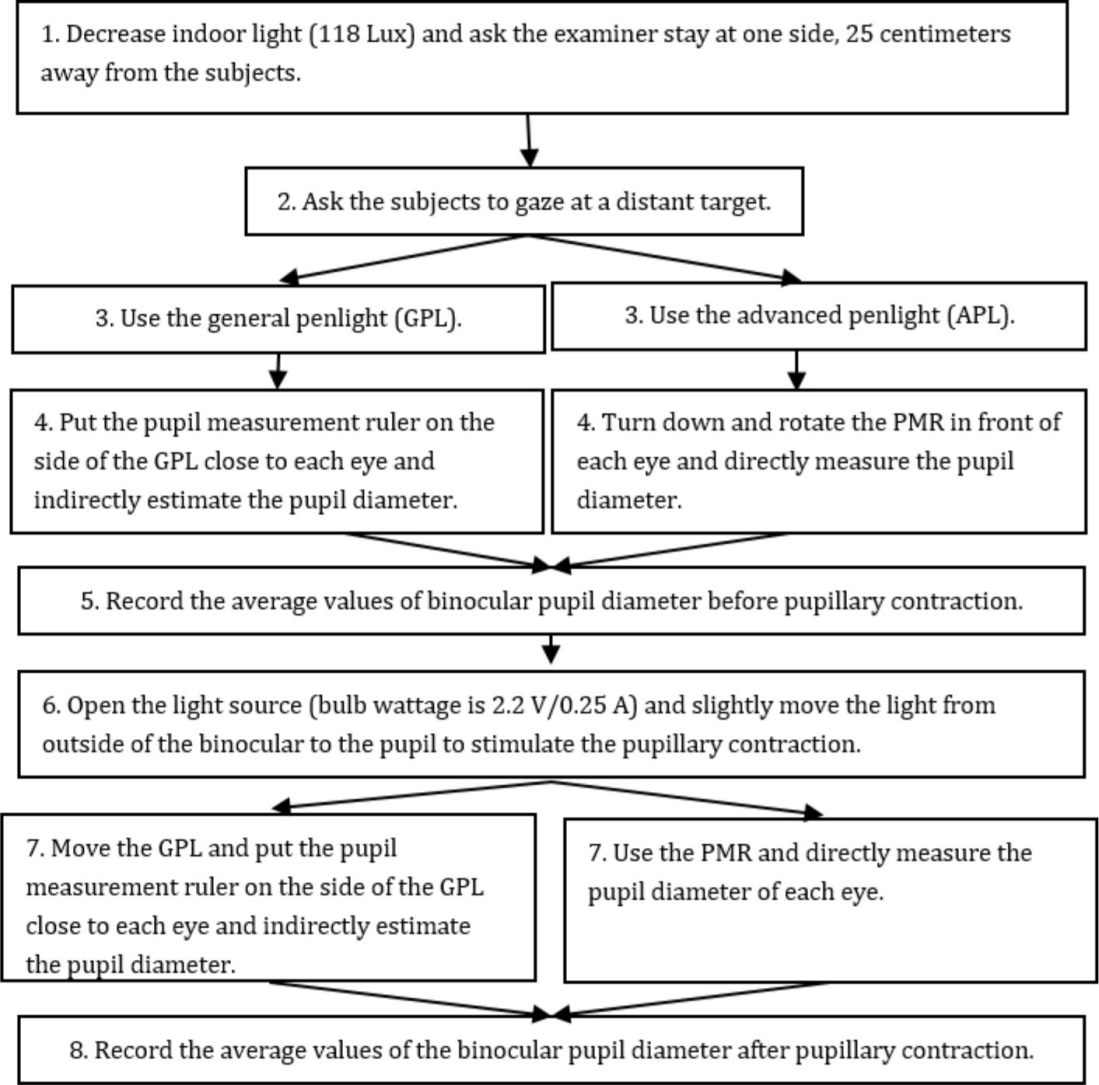
Eight steps of the standard procedures for the GPL and the APL.

One-group post-test and single-blind study designs were used in this study. The research was approved by an institutional review board (17MMHISO41e). The research was carried out during August 2017 to January 2018. Purposive sampling was used to recruit ninety nursing students from a college in northern Taiwan. The incursive criteria were nursing students who had experience operating a penlight in their nursing internship. The exclusive criteria was having serious eye disease affecting the eyesight or feeling anxious or panic in darker environments. We recruited the participants through leaflets. The participants who were willing to join the research could actively connect to researchers and arrange available times for the experiment. After the researcher explained the research purpose, process, possible benefit, and injury, all participants signed a written consent form. We asked the guardians or parents to sign a consent from for the participants who were under 20 years old. The standard pupil diameter was measured for each subject using a refractometer (RM) (Topcon Auto Kerato- refractometer; 75–1, Hasunuma-Cho, Itabashi-Ku, Tokyo, 174–8580, Japan), and the average values were calculated. The ninety participants measured the subject’s pupil diameter separately by the GPL and the APL using a standard eight-step procedure [18–19] (Fig 3).

The specification of the GPL is Spirit brands, model CK-907D, with a 3.0 V LED bulb lamp. The lux of ambient lighting had been measured in each operation and kept at 118 lux. It was suggested to take at least a 1 minute break between each light stimulation [18–19]. In this research, a 10 minutes break was used to restore the sensitivity of the subject's pupil for light stimulation. The pupil diameters before and after pupillary contraction were recorded separately for each participant. We defined pupillary contraction as the maximal constriction after light stimulation. The average times to perform the eight steps when using the GPL and the APL were also computed and recorded without informing the participants to avoid the Hawthorne effect. The participants were required to complete a questionnaire to ask for their opinion after the experiment.

### Estimation of sample size

G-power was used to estimate the sample size. On the basis of a power, effect size, and sample size of 0.95, 0.5, and 0.05, respectively, the sample size was calculated to be 45. At a loss rate of 20%, the sample size was calculated to be at least 54.

### Questionnaire of using opinion

A questionnaire was created to investigate the opinions of the participants for using the APL and the GPL. A four point Likert scale was used with 1 point means strongly disagree, 2 points disagree, 3 points agree, and 4 points strongly agree. Higher scores represent a more positive opinion. The Cronbach’s alpha coefficient of the questionnaire was 0.82 as determined by 30 pretest participants. The validity of questionnaire was evaluated using content validity by three experts. One nurse who has 7 years of experience working in the ICU, one assistant professor who majored in nursing, and one medical physician. The scores of content validity were four points (1 point means strongly disagree, 2 point means disagree, 3 point means agree, and 4 point means strongly agree) to evaluate the correctness, feasibility, appropriateness, and completeness of each question. The average scores of expert validity were between 3.7 to 3.9 points and represent a good validity.

### Analysis methods

The collected data was analyzed by using SPSS for Windows, version 20.0 (IBM Corp. in Armonk, NY). Descriptive statistics were used to analyze the basic characteristics of the participants. The mean differences in pupil diameter and operation time of the GPL and the APL measurements were analyzed by using the t-test. Bland-Altman plots and one sample t-test were used to find a potential dependency between means and differences within the GPL, the APL and the RM. The comparison of participants' opinions after using the APL and the GPL were performed by independent t-test.

## Results

### Characteristics of the participants

There were ninety participants, of whom 83% were female senior nursing students. The mean age of the participants was 20.01 (SD = 0.47) years. Approximately 78% (N = 70) of the participants had uncorrected visual acuity between 0.5 and 0.9. The mean period of internship in clinical nursing care was 7.12 (SD = 0.48) months. The mean frequency of using the GPL during the internship was 52.31 times (SD = 8.2). Approximately 88.0% of the participants acknowledged that the design quality of the penlight is important for disease progression monitoring (Table 1).

**Table 1:** Characteristics of the participants.

Variable	N (90)	%
Gender		
Male	15	17%
Female	75	83%
Age		
≤18	8	9%
19 ~ 20	72	80%
≥21	10	11%
Uncorrected visual acuity		
≥1.0	9	10%
≤0.9~0.5≥	70	78%
≤0.4	11	12%
Internship of clinical nursing care		
2~3 months	8	9%
4~5 months	12	13%
≥ 6 months	70	78%
Frequency of using a penlight during the internship		
≤40	9	10%
41~59	66	73%
≥60	15	17%
Recognized that good design of the penlight is important in disease progression monitoring for patients		
Yes	79	88%
No	11	12%

### Comparison of the GPL and the RM

Table 2 shows that the left pupil diameter before pupillary contraction (LPD BPC) was 1.52 mm larger than after pupillary contraction (APC) when using light stimulation by the GPL. The right pupil diameter before pupillary contraction (RPD BPC) was 1.47 mm larger than APC when using light stimulation by the GPL. The LPD BPC was 2.23 mm larger than APC when using light stimulation by the RM. The RPD BPC was 1.87 mm larger than APC when using light stimulation by the RM. The average values of pupil diameter measured by the RM were significantly larger than that of pupil diameter measured by the GPL before and after pupillary contraction. The results indicated the pupil diameters measured by GPL and the RM were different.

**Table 2:** Comparison of the GPL and the RM (N = 90).

	Average value of pupil diameter (mm)					t value	p value
	GPL (A)		RM (B)		(B)-(A)		
	M	SD	M	SD			
LPD BPC	4.33	0.92	5.56	0.25	1.23	12.36	<0.001
LPD APC	2.81	0.68	3.33	0.13	0.52	7.22	<0.001
RPD BPC	4.33	1.02	5.41	0.37	1.08	9.53	<0.001
RPD APC	2.86	0.71	3.54	0.27	0.68	8.24	<0.001

LPD = left pupil diameter, BPC = before pupillary contraction, APC = after pupillary contraction, RPD = right pupil diameter, GPL = general penlight, RM = refractometer, M = mean, SD = standard deviation

### Comparison of the APL and the RM

Table 3 reveals that the LPD BPC was 1.86 mm larger than APC when using light stimulation by the APL. The RPD BPC was 1.86 mm larger than APC when using light stimulation by the APL. The LPD BPC was 2.23 mm larger than APC when using light stimulation by the RM. The RPD BPC was 1.87 mm larger than APC when using light stimulation by the RM. There were no significant differences in the average values of pupil diameter as measured by the APL and the RM. The results indicated the pupil diameters measured by APL and the RM were very close.

**Table 3:** Comparison of the APL and the RM (N = 90).

	Average value of pupil diameter (mm)						
	APL (A)		RM (B)		(B)-(A)	t value	p value
	M	SD	M	SD			
LPD BPC	5.35	1.09	5.56	0.25	0.21	-1.87	0.065
LPD APC	3.49	0.69	3.33	0.13	-0.16	1.81	0.072
RPD BPC	5.33	0.87	5.41	0.37	0.08	-0.843	0.402
RPD APC	3.47	0.74	3.54	0.277	0.07	-0.411	0.682

LPD = left pupil diameter, BPC = before pupillary contraction, APC = after pupillary contraction, RPD = right pupil diameter, APL = advanced penlight, RM = refractometer, M = mean, SD = standard deviation

### Analysis of Bland-Altman plot

**Fig 4:**
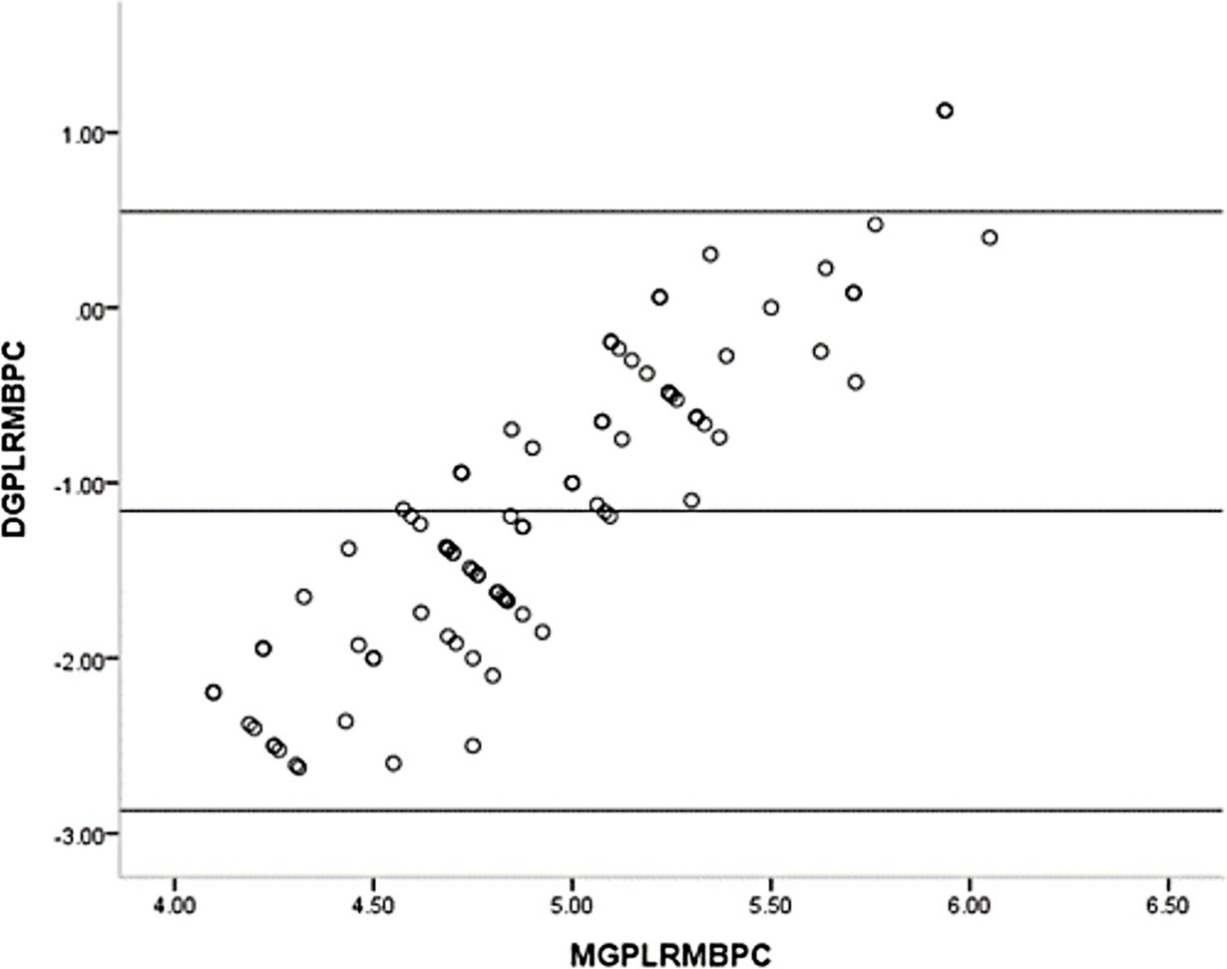
Bland-Altman plot of the GP and the RM before pupillary contraction. GP = general penlight; RM = refractometer; DGPLRMBPDC = difference of general penlight and refractometer before pupillary contraction; MGPLRMBPC = mean of general pen light and refractometer before pupillary contraction.

**Fig 5:**
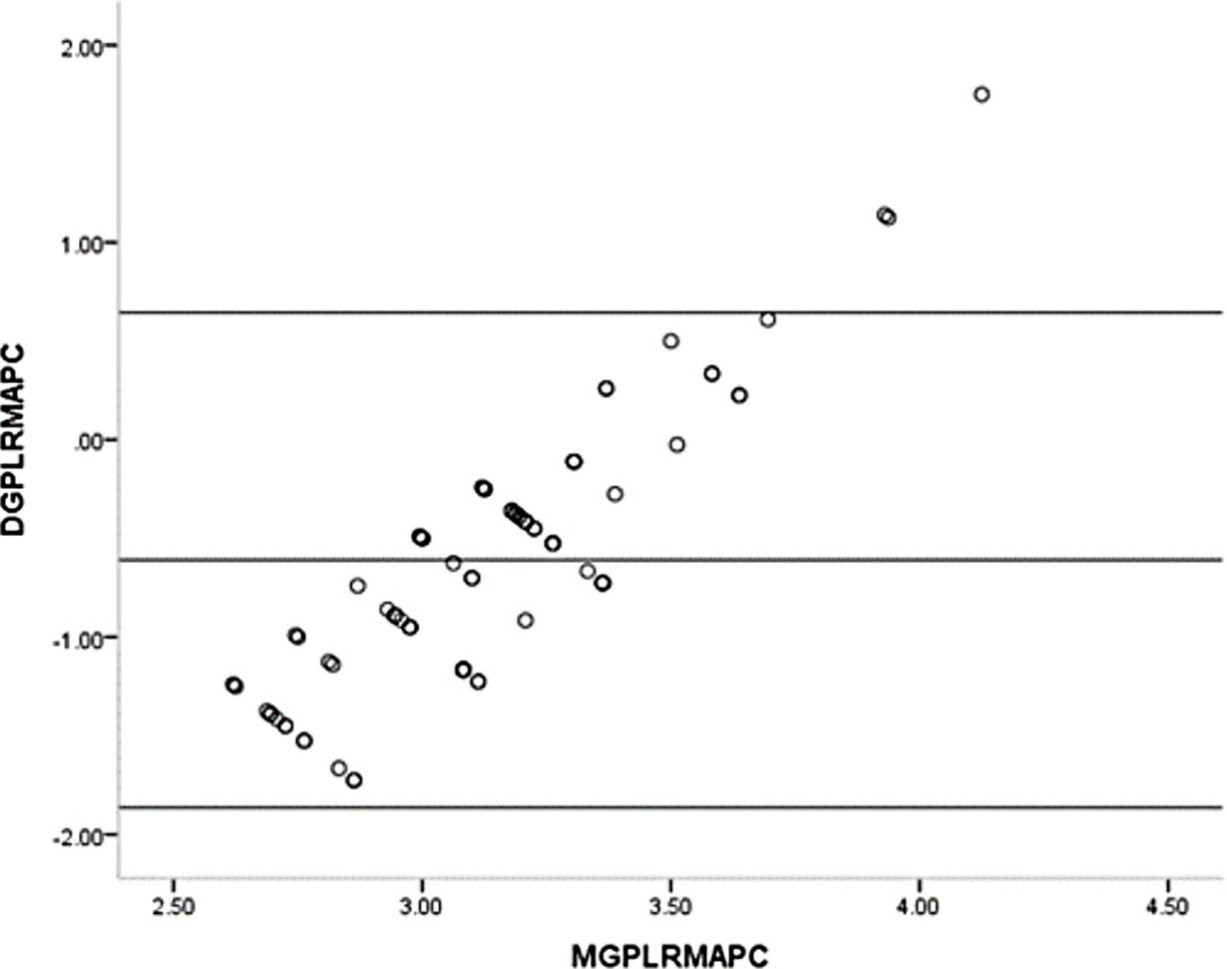
Bland-Altman plot of GP and the RM after pupillary contraction. GP = general penlight; RM = refractometer; DGPLRMAPC = difference of general penlight and refractometer after pupillary contraction; MGPLRMAPC = mean of general penlight and refractometer after pupillary contraction.

**Fig 6:**
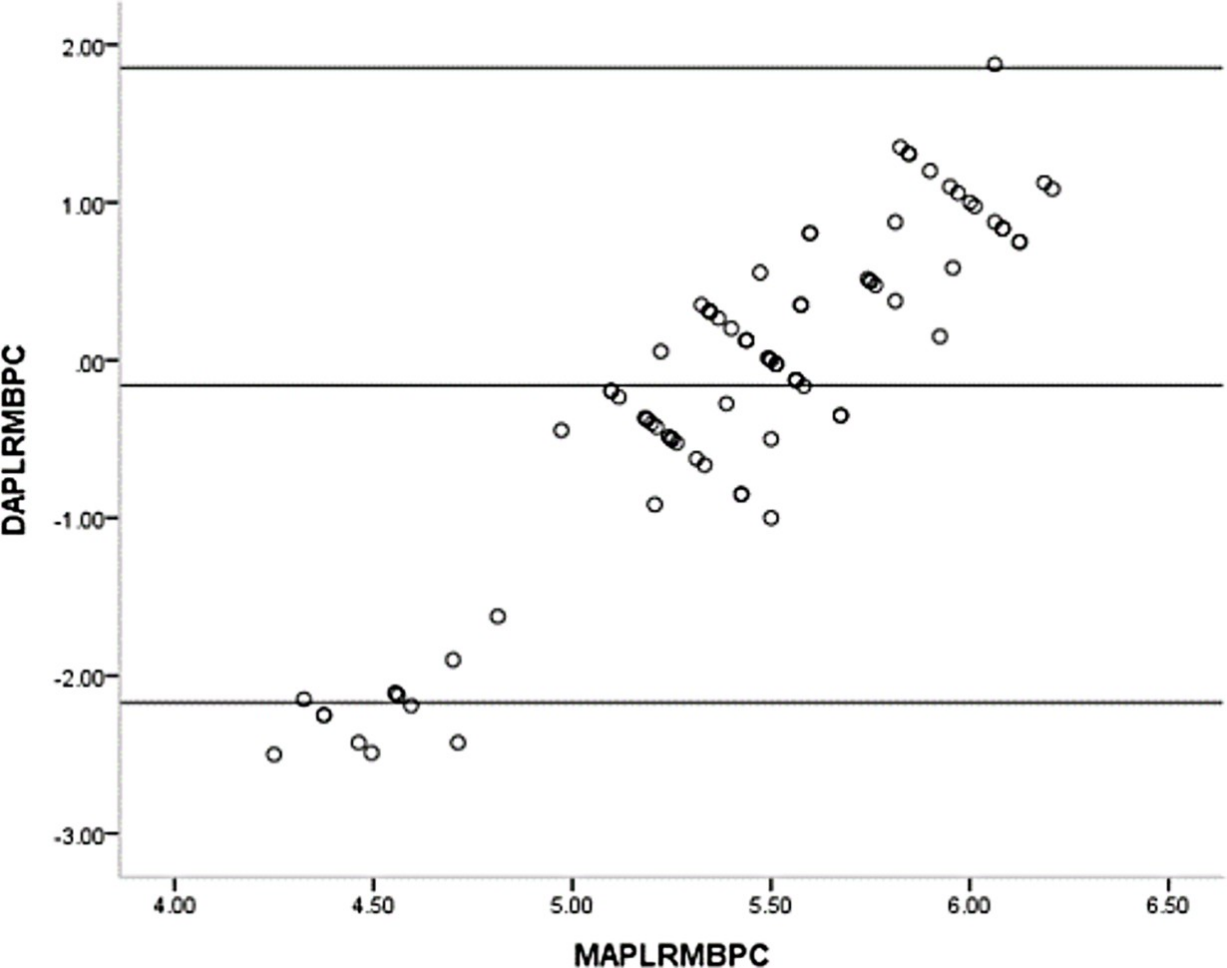
Bland-Altman plot of the APL and the RM before pupillary contraction. APL = advanced penlight; RM = refractometer; DAPLRMBPC = difference of advanced penlight and refractometer before pupillary contraction; MAPLRMBPC = mean of advanced penlight and refractometer before pupillary contraction.

**Fig 7:**
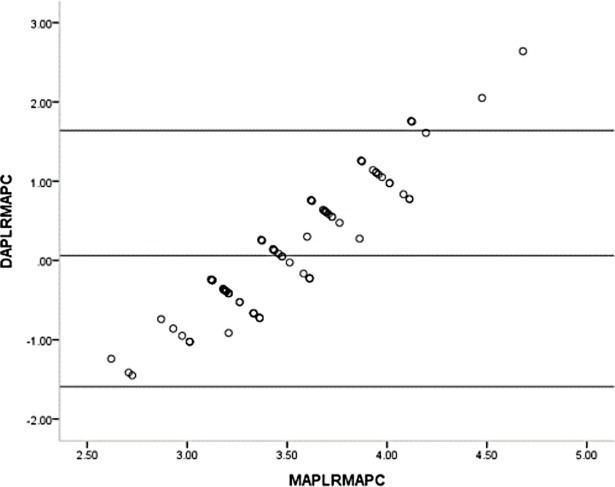
Bland-Altman plot of AP and the RM after pupillary contraction. APL = advanced penlight; RM = refractometer; DAPLRMBPC = difference of advanced penlight and refractometer after pupillary contraction; MAPLRMBPC = mean of advanced penlight and refractometer after pupillary contraction.

One sample t-test showed the mean differences of the GPL and the RM before (t = 12.626, p<0.001) and after (t = 9.028, p<0.001) pupillary construction reached the statistically significant differences. The mean differences of the APL and the RM before (t = 1.481, p = 0.142) and after (t = 0.712, p = 0.487) pupillary construction had no statistically significant differences. Bland-Altman plot of the GPL and the RM before and after pupillary construction presented in Figs 4 and 5 and revealed the significant differences. Bland-Altman plot of the APL and the RM before and after pupillary construction presented in Figs 6 and 7 and revealed no significant differences.

### Comparison of operational time for the APL and the GPL

The operational times (seconds) of eight standard procedures (Fig 3) performed with the GPL and the APL were measured separately. The average operational time of the eight steps performed with the GPL was 14.81 seconds. The average operational time of the eight steps performed with the APL was 6.12 seconds, which was 8.72 seconds shorter (t = -3.81; p = 0.001) than that of the GPL (Table 4).

**Table 4:** Comparison of operational time of the GPL and the APL (N = 90).

	GPL (A)		APL (B)		(B)-(A)	t value	p value
	M	SD	M	SD			
Operational time (seconds)	14.84	1.43	6.12	0.91	-8.72	-3.81	0.001

APL = advanced penlight, GPL = general penlight, M = mean, SD = standard deviation

### Comparison of participants' opinions regarding the use of the APL and the GPL

The average scores of the five questions were all significantly higher for those using the APL than those using the GPL (Table 5). In the item of the confidences to judge the pupil diameter, the mean difference was 1.23 (t = 11.85; p<0.001) higher in the APL than in the GPL. The mean difference between in the item of reduced duration to judge the pupil diameter with the APL and GPL was 1.13 (t = 9.67; p<0.001). All of the participants considered that the convenience and confidence of pupil diameter measurement were higher when using the APL rather than the GPL, and therefore, were more inclined to use the APL.

**Table 5:** Comparison of participants' opinions of using the APL and the GPL (N = 90).

	APL(A)		GPL(B)		(A)-(B)	t value	*p* value
	M	SD	M	SD			
The easiness to observe the value of the pupil diameter	3.41	0.51	2.35	0.49	1.06	11.6	<0.001
The reduced duration to judge the pupil diameter	3.43	0.52	2.3	0.54	1.13	9.67	<0.001
The accuracy to judge the value of the pupil diameter	3.47	0.51	2.39	0.53	1.08	8.76	<0.001
The confidence to judge the pupil diameter	3.59	0.54	2.36	0.71	1.23	11.85	<0.001
The willingness to use the tool	3.68	0.51	2.74	0.49	0.94	9.49	<0.001

APL = advanced penlight, GPL = general penlight, M = mean, SD = standard deviation

## Discussion

In our study, the mean values of pupil diameter measured by the GPL had significant differences from the standard values measured by the RM. The result was similar to Couret, et al. [20], compared to hand-held electronic monocular pupilometer, the standard measurement in pupil size and pupil reflex by penlight yields inaccurate data. Although, the electronic and automated pupillometry is a more reliable instrument in patients' pupil assessment, the GPL is still frequently used in clinical care situations to measure the pupil diameter and pupil reflex of the autonomic nervous system. It is worthwhile to improve the function of GPL in the most cost-effective way to improve the accuracy of measuring the pupil diameter during pupil reflex.

The mean values of pupil diameter measured by the APL were much closer to the standard values measured by the RM. Previous research had discovered that by using the penlight with a gauge could improve the result consistency of pupil size assessment than without using a gauge [21]. In the APL, the gauge had been redesign into a moveable PMR to directly compare the pupil diameter. Therefore, the consistency and accuracy of pupil diameter measurement could be enhanced. In addition, the convenience of saving time and accuracy measure design of APL met the expectations of participants with nursing back grounds. It could be applied for health and nursing care practitioner in pupil assessment and disease monitoring in the future. In the result, the average values of the pupil diameter measured by the RM were larger than that of the GPL and APL before and after pupillary contraction. The change in the pupil diameters were influenced by the state of both eyes and degree of retinal illumination [22–23]. The eyes of the subjects are very close to the RM during measurement. The light sensors of the pupil decreased and lead to pupil dilation, which may account for the larger values of pupil diameter in our measurements. A visually direct measurement method by the APL can significantly improve the accuracy of pupil diameter measurements. The design of the PMR allows it to be placed very closely above the pupil so that the examiner can see through the scale to accurately estimate the value of pupil diameter before and after pupillary contraction without moving the APL. Therefore, the values of pupil diameter measured by the APL were all significantly correlated with the values measured by the RM.

Pupil diameters before and after pupillary contraction change within a few seconds. The differences between the average values of the pupil diameters before pupillary contraction were 0.21 mm and 0.08 mm in the left and right eyes, respectively, for the RM and the APL measurements. The differences between the average values of the pupil diameter after pupillary contraction were -0.106 mm and 0.07 mm in the left and right eyes, respectively, for the RM and the APL measurements. The differences between the average values of the pupil diameter in same eye measured were 1.23 mm, 1.08 mm, 0.52 mm, and 0.68 mm in the RM than the GPL. The design of the APL allows for detection of a slight change in the pupil diameter and the resulting measurements were closer to the values as measured by the RM.

The operation time was 8.72 seconds shorter in using the APL compared to the GPL. The first reason for this is that operating the APL does not require moving the pupil measurement ruler located on the side of the GPL close to each eye. The second reason is that the direct measurement of the APL can increase the users’ confidence in the values of pupil diameters and decrease the time in repeated measurement and interpretation. So far, no research was conducted to study the influence of performance confidence and operational time on the pupil diameter measurements. Our results indicate that the confidence in the values of pupil diameters can significantly decrease the operation time for measurement. Studies rarely compare the users' subjective views of different pupillometry methods. In our results, the mean difference in the questionnaire answers can be interpreted as an effect of physiological parameters. For example, the mean difference of the item in the accuracy to judge the pupil diameter was 1.08 (t = 8.76, p<0.001) higher when using the APL than the GPL. These results are consistent with the results of pupil diameter measurements by the APL and the RM.

Compared to the GPL, the APL has a more innovative design and evaluation method of pupil diameter measurement. Its cost is lower. We estimate the price of the APL to be approximately US $9.5. Moreover, the APL can be handmade using available raw materials. The production technology is easy. The potential for mechanization is high, which is beneficial for future mass production. The APL is easy to use and no extra training is required. Pupillometry can be used in many studies and applications. In addition to the evaluation of nursing care quality, medical diagnosis, and disease prognosis, changes in the pupil diameter could become a method of understanding and measurement of cognitive acts [24]. So far, there is no similar product. Therefore, there will be no competition on the market and the potential market is large. At present, general wards, intensive care units, and emergency rooms are equipped with GPL. Therefore, as long as the GPL is slightly modified and improved into the APL, it not only can achieve precise and confidence pupil diameter measurement, but also save the measure time for health and nursing care practitioners.

There are several limitations that need to be mentioned. First, due to the limitation of budget and instruments, we chose the RM which is used for ophthalmology to measure the standard pupil size. Thus, the illumination conditions are different and the resulting pupil sizes are different in penlight and the RM. Comparable device is thought to be handheld infrared device, such as, NPi-200 (NeurOptics). Its accuracy has already been proved and shows the temporal change of the pupil diameter stimulated by the LED light source. Second, the participants were purposively sampled and recruited from the same college, and most of them were female, which may have affected the results. To increase the generalizability of the results, we suggest that future studies be performed to determine the inclusive criteria by using gender-matched samples. Diversification of participants' recruitment sites is also needed. We only found the modified design of a conventional LED penlight with pupil gauge had significant differences from the GPL in statistics. However, we cannot ensure its clinical significance due to the fact that the subjects were in good health conditions. We suggest in the future experiment, the APL should be applied in patients with brain or eyes disease to confirm its clinical significance.

## Conclusions

Compared with the GPL, the average pupil diameter measured by the APL was closer to the standard pupil diameter measured by the RM. The average operational time for the APL was significantly shorter than that of the GPL. All of the participants believe that the convenience and confidence of the pupil diameter measurement were higher when using the APL rather than the GPL, and they were more inclined to use the APL in a clinical health and nursing care setting. The use of the APL can significantly enhance the accuracy and efficiency of pupil diameter measurements.

## Supporting information

S1 Consent (DOCX)
