# Supplementary material for: The effect evaluation of advanced penlight
Source: PLoS One. 2018 Nov 7;13(11):e0205978. doi: 10.1371/journal.pone.0205978 (PMC6221280; doi:10.1371/journal.pone.0205978)
Supplement: S1 Consent — (DOCX) [file pone.0205978.s001.docx]

**Supporting Information**

**English Version of Consent Form**

依據100年12月28日公布施行之人體研究法

(華總一義字第10000291401號令公布)

Human subject (or subject) consent form

| Research Project Name: Exploring the Accuracy of Advanced Penlight (APL) for Improving Pupil diameter Measurement |
| --- |
| Executive unit: Mackay Junior College of Medicine, Nursing, and Management  Entrusted unit / pharmaceutical factory: none  Main moderator: Chih-Yin Chien Title: Lecturer Phone: 0935212328  Co-host: Piao-Yi Chiou Title: Assistant Professor Phone: 0935883372  24-hour emergency contact and telephone: Jane Yin 0932512328 |
| Name of the subject (or subject): Subject number:  Gender: Date of birth:  Medical record number:  National ID card uniform number:  Mailing address:  Contact number:  Legal representative / name of consent holder:  Relationship with the subject (or subject):  Gender: Date of birth:  National ID card uniform number:  mailing address:  Phone: |
| 1. Introduction  The measurement of the size of the pupil and the reactive response are often seen by clinical professionals as "window to view the brain" (Watefhouse, 2005). The evaluation and measurement of pupils can be used as an indication of treatment and prognosis of brain disease. The results of pupil diameter measurement are quite significant for clinical care situation. Therefore, it is important to improve the convenience and accuracy of operation process of the penlight to improve quality of patient care. The use of general penlight (GPL) usually needs a pupil measurement ruler for assessing the size of the pupils. However, critical conditions often occur instantaneously, such as head impact and sudden changes in consciousness. If the medical staff forgets to carry the pupil measurement ruler, it will take more of time to take the pupil measurement ruler from nursing station and delay the immediate assessment, and also reduce the efficiency of the action. Even if there is a sticking pupil measurement ruler on the side of the GPL. The pupil diameter only can be measured indirectly by closing the measurement ruler on the GPL to the eyes. To evaluate the size of the pupil after the reaction, the GPL needs to be removed after the pupil contraction. In fact, the resulting value does not represent the size of the pupil when it is contracted, and moving the GPL also extends the evaluation time. In view of the above problems of pupil measurement, the aim of the research is to improve the function of GPL of "non-indirect comparison" to promote the accurate, convenient, and rapid in operation by "direct comparison" during the contracted of the pupil. The result could improve the accuracy of the assessment. |
| 2. The Purpose of This Study  (1) Compare the difference in the accuracy of pupil measurement between the general penlights (GPL) and advanced penlight (APL).  (2) Compare the difference of pupil measurement time between the GPL and APL.  (3) Compare the view difference between the participants after using the GPL and APL. |
| 3. The Main Inclusion and Exclusion Criteria of The Study Subject:  (1) Mainly included conditions: senior students in the nursing department, who have completed the physical assessment course, have the academic and technical basis of pupil assessment, and have experience in clinical nursing internship care. A person under the age of 20 must sign a consent form with both the subject and the legal representative.  (2) Exclusive conditions: Non-nursing students, who have not completed a physical assessment course, have no pupil assessment knowledge and real experience, and have no clinical nursing internship experience. |
| 4. Research Object Method and Related Test:  After obtaining the participants’ consent form, a automatic computer of refractometer (RM) is used to obtain the standard pupil size of the subject. After the researcher explains the operation process of the GPL, the participants use the GPL to measure the diameter of the subject's pupil before and after light reaction. Then, the researcher explains the operation of the APL. The same participant measures the pupil diameter of the same subject by APL. The measurements time are counted and recorded without notify the participants. The participants have to fill out the questionnaire after the measurement operation. It is estimated that each participant need for 20-30 minutes to complete the whole process. |
| 5. Possible adverse reactions, incidence, and remedies for damage:  The possible adverse reactions in the study were the emotional response such as anxiety of the participants caused by stress during the measurement process. The pupils were repeatedly tested and caused eye discomfort of the subjects. Therefore, at the beginning of the study, the researcher provided the appropriate description of research process for the participants and gave sufficient operation time to alleviate the anxiety of the participants. In addition, each time the pupils are measured, the subject's eyes are rested for at least 1-3 minutes to restore the pupil's photoreaction and allow the subject's eyes to rest properly. |
| 6. Other Possible Treatments and Instructions:  N/A |
| 7. Expected Test Results( Human trial or clinical study applicable to medical law):  N/A |
| 8. The Contraindications, Limitations and Copings of the Subjects During the Trial:  Subjects with eye problems are not eligible for this study. |
| 9. Confidentiality of Subject’s Personal Data:  The participant’s data are identified by number, and the data is encoded to remove all personally identifiable and linked data, all of which are locked for privacy. All research materials can only be reviewed by the moderator, monitor, co-host, auditor, and the human research ethics review committee, and the government authorities. |
| 10. The Retention Period, Application Planning and Reuse of the Subject's Biological Specimen, Personal Data or Its Derivatives:    This study does not produce biopsies, and the retention period of personal data is 5 years. |
| 11. Compensation related compensation (compensation) compensation or insurance mechanism:   - If the trial is carried out according to the clinical trial plan set by the Institute, and the adverse reactions (including the expected risks and side effects) will cause damage, the Mackay Junior College of Medicine, Nursing, and Management will be responsible for damages and all medical expenses. However, the subject agrees that the adverse reactions (including expected risks and side effects) already recorded in the consent form will not be compensated or any other compensation. (Note: If there is no sponsorship from the pharmaceutical company, please fill in the testing agency for the trial client) - The Mackay Memorial Hospital is willing to provide professional medical care and medical advice if the trials are carried out in accordance with the clinical trial plan of the Institute and the damage caused by adverse reactions (including expected risks and side effects) occurs. You do not have to bear the necessary medical expenses for adverse reactions (including expected risks and side effects). - Except for statutory compensation and medical care, this study does not provide other forms of compensation or compensation. If you are unwilling to accept such risks, do not participate in the trial. - You will not lose other legal rights as a result of signing this consent form. - If you already have other medical-related, health-related commercial insurance or disability insurance, joining this clinical trial may affect the rights of your existing commercial insurance. This trial cannot guarantee the extent to which your rights in this section are affected. - There is no insurance coverage insurance for this study. |
| 12. Subject rights:   - 1. Any major findings related to your health or illness that may affect your willingness to continue clinical trials will be provided to you immediately.   2. If you have doubts about the nature of the work during the test, and if you have any opinion or suspected of being a victim of the right to participate in the study, you can contact the Human Research Ethics Review Committee of the hospital via a telephone number: (02) 2543-3535 to 3486~3488.   3. In order to carry out the research, you must accept the explanations and instructions of the lecturer of . If you have any questions or problems at the time of the trial or during the trial, you can contact Chih-Yin Chien who is an instructor of the department of Nursing, Mackay Junior College of Medicine, Nursing, and Management.   4. The physician has given you a copy of the consent form and has fully explained the nature and purpose of the study and the contents of this consent form. Your doctor has answered your questions about medicines and research.   5. The study may derive the commercial benefits and the application of the agreement: the results of this study are mainly based on the publication of the work, there are no derivative commercial interests and agreements, and there are no derivative commercial interests and agreements with the subjects. |
| 13. Exit and Suspension of the Trial:  You are free to decide whether or not to participate in the trial; you may withdraw your consent at any time during the trial, and withdraw from the trial without any reason, and will not cause any unpleasantness or affect the medical care of your physician in the future. The trial host or sponsor may also suspend or terminate the trial if necessary. |
| 14. signature   - 1. The main moderator and co-host have explained in detail the nature and purpose of the above research methods in this test plan, and all the contents of this consent form.   Signature of the main moderator/co-host:  Date:   - 1. The subject has been thoroughly informed of all the contents of this consent form above, and the questions about the test plan are explained in detail by the project leader. I agree to be a voluntary subject for this clinical trial program.   Subject signature:  Legal agent signature:  Relationship with the subject (or subject):  Date:   - 1. I agree to be a voluntary subject for this clinical trial program, and signatures with fingerprints, crosses or other symbols must be signed by two people to make the subject's signature equal. (This field can be decided by the trial host to increase the witness signature and its number)   a. Witness signature:  Date:  b. Witness signature:  Date: |

Bellowing are the descriptions of the signature. Please refer to the narrative of the text, and pay attention to the scope of the nearest relative agent.

(1) According to Article 12 of the Human Research Act:

Subjects were excluded from the fetus or corpse, and were limited to adults with meaningful abilities. However, research is benefit for specific population group or cannot be replaced by other research subjects are not limit to above description. When the subject is a fetus, the first consent shall be made by the mother; for the person with limited capacity or the person who is assisted by the declaration, the consent of the person and the legal representative or the assistant shall be obtained; for the incapacitated person or the person subject to custody shall obtain the consent of his or her legal representative or guardian; for the first adult of the proviso, the consent of the person concerned shall be obtained in the following order:

1. the spouse.

2. adult children.

3. parents.

4. brothers and sisters.

5. grandparents.

In accordance with the written consent of the person concerned in the preceding paragraph, the written consent of the person concerned can be done by one person; when the meaning of the relationship is inconsistent, the order of the preceding paragraphs is determined. The person in the same order of the preceding paragraph is the first to be close, the opposite is the first, the relatives of the cohabitation are the first, the relatives who are not living together, and the senior is the first.

Applicable medical law human test case: According to Article 5 of the Human Body Management Regulations:

Subjects who are recruited in accordance with the first paragraph of Article 79 of the Medical Law or who have been married to a minor, the moderator shall obtain the consent of the person concerned in the following order:

First, the spouse.

Second, parents.

3. Adult children living together.

4. Grandparents living with the subject.

5. Brothers and sisters living together with the subjects.

6. Other relatives of the fact of cohabitation in the most recent year.

The consent of the person in question of the preceding paragraph shall not violate the meaning expressed by the subject.
